# Evaluation of prophylactic and therapeutic activity of camel whey protein and its trypsinized hydrolysate against experimental trichinellosis

**DOI:** 10.1186/s12906-025-05083-7

**Published:** 2025-09-22

**Authors:** Sara A. Abdel Salam, Aisha El-Attar, Marwa Abdelaziz, Hoda A. Rashed

**Affiliations:** 1 Faculty of Medicine, Department of Medical Parasitology, Alexandria University, Alexandria, Egypt; 2 Faculty of Agriculture, Department of Dairy Science and Technology, Alexandria University, Alexandria, Egypt; 3 Faculty of Medicine, Department of Pathology, Alexandria University, Alexandria, Egypt

**Keywords:** *Trichinella spiralis*, Prophylactic, Therapeutic, Camel whey protein, Tryptic hydrolysate, Antioxidant potentials

## Abstract

**Background:**

To date, control of trichinellosis has been dependent on the conventional chemotherapeutic agent, albendazole (ALB), with its hitherto unsolved flaws, including a serious adverse profile, minor activity against muscle larva (ML) stages, and resistance development. Camel whey protein (CWP), a unique non-pharmaceutical nutraceutical, has altered the treatment trajectory of a plethora of pathological conditions. The present study aimed to evaluate the prophylactic and therapeutic profile of CWP and its tryptic hydrolysate (TWH) against experimental intestinal and muscular trichinellosis.

**Methods:**

CWP and TWH were characterized by free amino acids analysis using the HPLC Pico-Tag method and DPPH free radical scavenging antioxidant assay. 200 µl of five-day oral dosing of CWP and its TWH was prophylactically and therapeutically administered to mice. Parasitological, ultrastructural, biochemical, and histopathological studies were performed for assessment of their anti-trichinella activity. Preclinical data were subjected to analysis of variance and a Tukey post hoc test for pairwise comparisons.

**Results:**

The free amino acids profile and high DPPH scavenging antioxidant activity of CWP (79.2%) and TWH (82.7%) were determined. Parasitologically, TWH showed the highest prophylactic (84.0%R and 83.8%R) and therapeutic (94.6%R and 93.9%R) activity in mean worm count recovered from intestinal and muscular stages, respectively. Lesser reductions were recorded by CWP, whether prophylactically-treated (73.9%R and 72.1%R) or therapeutically-treated (84.5%R and 83.9%R) against aforementioned stages, respectively. In addition, scanning electron microscopy revealed that the most severe ultrastructural deformities were observed in TWH-treated worms. Biochemically, the prophylactic and therapeutic administration of TWH recorded the highest antioxidant serum level of reduced glutathione (GSH) that curbed the oxidant malondialdehyde (MDA). Histopathologically, TWH ameliorated the intestinal and muscular pathology compared to CWP.

**Conclusions:**

To the best of our knowledge, this is the first study on the prophylactic and therapeutic administration of CWP and TWH against experimental trichinellosis, showing the superiority of TWH in inducing multistage activity and ameliorating the intestinal and muscular pathology.

## Background

Trichinellosis is a ubiquitous emerging or remerging meat-borne zoonotic disease caused by the polytypic genus of *Trichinella*. Among the extant species, *T. spiralis* is the most common species infecting humans [1]. The course of trichinellosis is divided into enteral, muscle invasion, and convalescent stages [2]. About 10,000 cases of human infections with trichinellosis have been reported annually from 55 countries, with a 0.2% mortality. Death occasionally develops as a sequelae of myocarditis, and less frequently encephalitis, vascular thrombosis, bronchopneumonia, and sepsis [3].

Benzimidazoles, albendazole (ALB) and mebendazole, are the mainstay of treatment for trichinellosis [2]. Despite their pronounced efficacy against intestinal adult worms, they are ineffective against ML encysted in striated myocytes [3, 4]. Additionally, poor water-solubility, low bioavailability, serious side effects, as well as emerging resistance of benzimidazoles, are soliciting concerns about their therapeutic utility [5]. Despite intensified control strategies exerted to prevent the acquisition of *T. spiralis* infection, the complexity of the parasite remains a major hurdle hindering the effectiveness of vaccination [2]. To overcome this embarrassing situation, exploiting complementary medicine as an alternative approach for the prevention and treatment of trichinellosis may provide a versatile, future plausible choice.

Camel milk, the white gold of the desert, is reputed as one of the most important traditional milks in arid and semi-arid regions [6]. Compared to milk of other ruminants, dromedary camel milk is rich in protective proteins, oligoelements, and vitamins (C and E) of high thermal stability as well as low sugar and fat content [7, 8]. It is commensurate with human mother’s milk in its antiallergenic protein profile, as it is rich in α-lactalbumin and lacks the milk allergen, β-lactoglobulin [9]. Besides the nutritional values of camel milk, several in vivo studies have investigated the robust antiparasitic therapeutic potential of camel milk against *Entamoeba histolytica* [10], *Giardia lamblia* [11], *Leishmania donovani* [12], *Toxoplasma gondii* [13], *Schistosoma mansoni* [14], and *Echinococcus granulosus* [15].

Camel milk whey protein (CWP) fraction is regarded as a gold mine of a plethora of highly stable bioactive peptides such as lactoferrin, α-lactalbumin, serum albumin, lactoperoxidase, lysozyme, lactophorin, immunoglobulins, and peptidoglycan recognition proteins [16]. It possesses high stability and antioxidant activities [17]. Apart from the exponential growth witnessed in applications of CWP fraction as hepatoprotective, antidiabetic [18] and anticancer therapeutic agents [19], it has been exploited against various pathogenic strains of bacteria and virus [20].

The release of different bioactive peptides by enzymatic hydrolysis of CWP using proteolytic digestive enzymes such as pepsin, chymotrypsin, and trypsin has revolutionized the biomedical field [21]. Intuitively, camel whey hydrolysate exhibits multifunctional bioactive properties with higher antimicrobial and antioxidant properties than pure peptides [22]. Besides the potent antihypertensive properties of camel whey hydrolysate [23], trypsin-hydrolysed camel whey (TWH) possesses antibacterial [22] and antifungal activities [24].

The ongoing insights of ALB, low efficacy against encysted larvae and resistance, highlight the way towards new anti-*Trichinella* agents. The multitude of nutritional and therapeutic benefits of CWP and its trypsinized hydrolysate have paved the way as a non-pharmaceutical natural agent. Thus, the objective of the current study was to evaluate their prophylactic and therapeutic effects against experimental enteral and muscular trichinellosis.

## Methods

### Separation of CWP and Preparation of TWH

Camel milk (4.23% fat and 3.47% total protein) was obtained from the Centre of Scientific Research in Marsa Matruh governorate. CWP was separated at the Dairy Science and Technology Department, Faculty of Agriculture, Alexandria, Egypt, according to Wang et al. [25]. Milk protein hydrolysate was prepared according to Otte et al. [26]. The hydrolysis process was initiated by adding 2 ml of trypsin solution to the protein solution and vortexed for 30 s. The mixture was incubated at 40 °C, and a 1 ml sample was withdrawn after 24 h of hydrolysis. Then, enzymatic inactivation was done by heating at 90°C for 15 min. Afterward, the mixture solution was cooled in an ice bath for 20 min, centrifuged at 10,000xg for 10 min, and the supernatant was used for further analyses [27].

### Characterization of CWP and TWH

#### Free amino acids analysis

The amino acid composition of CWP and TWH samples was analyzed using high-performance liquid chromatography (HPLC) Pico-Tag method (Waters Associates, Millipore Cooperative, USA). Pre-column derivatization of the samples’ amino acids was done using phenylisothiocarbamyl. The phenylthiocarbamyl derivatives were separated by reversed-phase gradient elution HPLC and detected by their ultraviolet (UV) absorbance at a fixed wavelength of 254 nm (2489 UV/Vis Detector) [28, 29].

#### Antioxidant activity assay

Free radical scavenging activity of CWP and TWH was measured by 1, 1-diphenyl-2-picrylhydrazyl (DPPH) [30]. Using a spectrophotometer (UV-VIS Milton Roy), the absorbance (A) was measured at 517 nm. Ascorbic acid was used as a reference standard compound, and the experiment was performed in triplicate. Using the log dose inhibition curve, the inhibitory concentration of 50% (IC_50_%) of DPPH free radical, was calculated. The percentage of DPPH scavenging effect was determined using the following equation:

$$\:\begin{aligned} &\text{DPPH}\:\text{scavenging}\:\text{effect}\:\%\\& \quad =\:\frac{\text{Acontrol}\:-\:\text{Asample}\:}{\:\text{A}\text{control}}\times100 \end{aligned}$$

### Animals and ethics statement

Laboratory-bred parasite-free Swiss albino male mice, aged 6–8 weeks and weighed 20–25 g, were obtained and retained in the animal house of Medical Parasitology, Faculty of Medicine, Alexandria University, Egypt, under standard housing conditions. Animals were housed, treated, and sacrificed according to the Egyptian national animal ethics guidelines. They were anesthetized by intraperitoneally injecting 40 mg/kg of sodium pentobarbital, and blood was collected from the jugular veins. Subsequently, the unconscious mice were immediately euthanized by cervical dislocation. The protocol was approved by the Ethical Committee of the Faculty of Medicine, Alexandria University, Egypt (protocol approval number: 0306391).

### Parasite and animal infection

*T. spiralis* strain was initially purchased from Theodore Bilharz Research Institute (Giza, Egypt) and maintained in the Medical Parasitology laboratory for sequential infection. On the 28th day post-infection (dpi), the whole eviscerated skinned carcass was minced, and infective ML were retrieved by the artificial digestion method. The minced muscles of the infected mouse were digested in 1% pepsin and 1% concentrated hydrochloric acid in 200 ml distilled water and swirled at 37 °C using a magnetic stirrer. After two hours, the mixture was sieved in a conical flask. Thereafter, the supernatant was discarded after allowing ML to sediment and they were collected and microscopically counted. For infection, 250 *T. spiralis* ML were adjusted in 0.1 ml and orally inoculated into the mouse using a gastric tube [31].

### Animal grouping and experimental design

Ninety-six mice were randomly allocated into 5 main groups. **Group I** included 6 non-infected non-treated mice. **Group II**, 18 non-infected treated mice were subdivided into 3 equal subgroups as follows: Subgroup IIa, non-infected CWP-treated; Subgroup IIb, non-infected TWH-treated; and Subgroup IIc, non-infected ALB-treated. **Group III** consisted of 12 *T. spiralis*-infected non-treated control mice. **Group IV** (Prophylactic group), 24 mice were subdivided equally as follows: Subgroup IVa, CWP-pretreated infected; and Subgroup IVb, TWH-pretreated infected. ** Group V** (Therapeutic group) included 36 infected treated mice that were subdivided equally into 3 subgroups as follows: Subgroup Va, infected CWP-treated; Subgroup Vb, infected TWH-treated; and Subgroup Vc, infected ALB-treated.

**Fig. 1:**
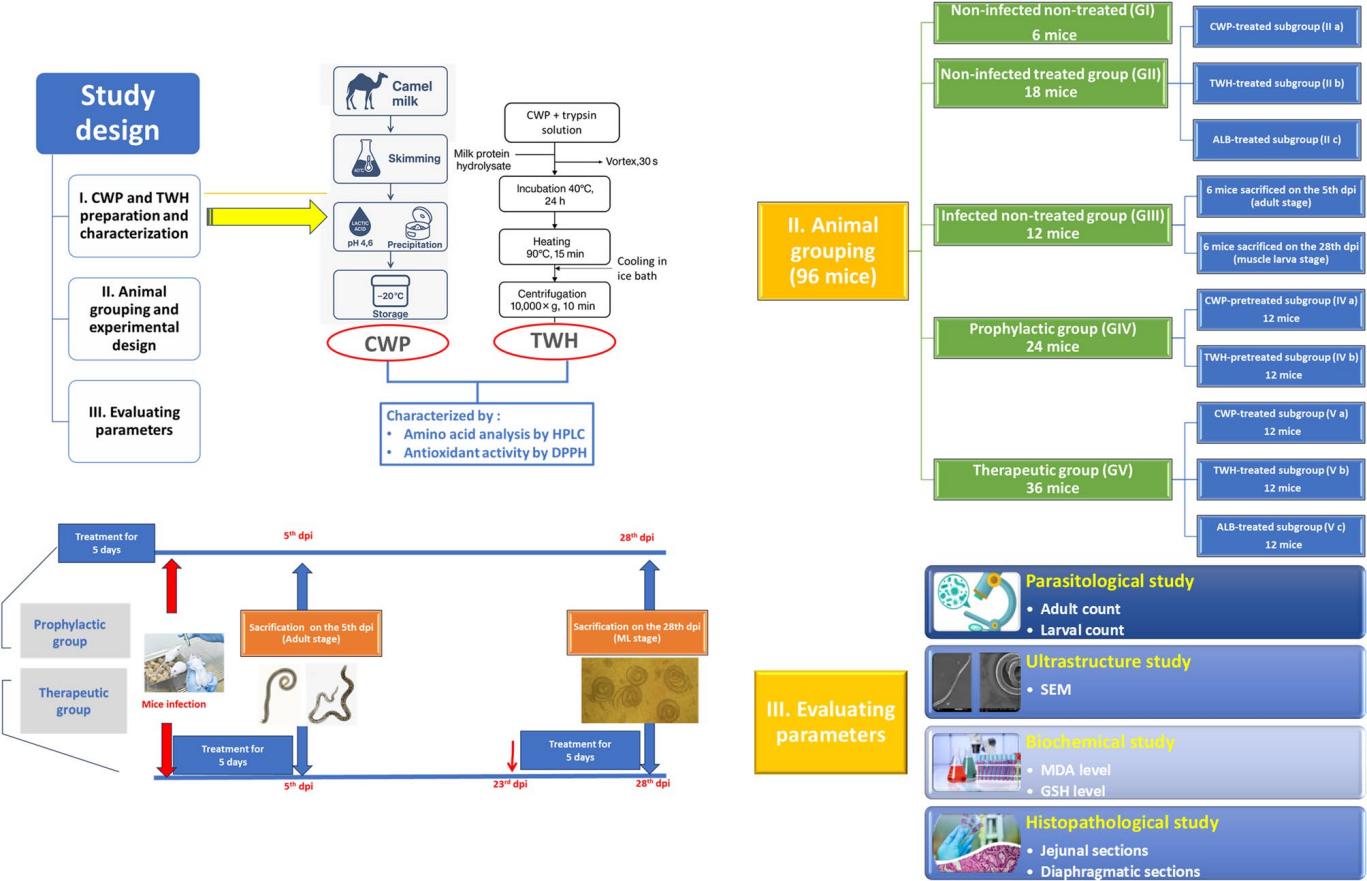
Infographic representation of the experimental study design, animal grouping, and treatment timelines

According to Maghraby et al. 2005, the calculated daily oral dose of CWP and TWH was 200 µl per mouse [14], while ALB was administered to mice at a dose of 50 mg/kg [32]. The treatments were administered for five consecutive days by gastric tube. In the prophylactic subgroups (IVa and IVb), all mice started treatments five days before infection. From each therapeutic subgroup (Va, Vb, and Vc), six mice initiated the five-day treatment on the same day of infection, corresponding to the adult stage of *T. spiralis* [33]. While the remaining six mice started the treatment course on 23rd dpi, corresponding to the timing of nurse cell formation in the muscular phase [34]. From each infected Group (III, IV, and V), six mice were euthanized at two different time points, namely on the 5th and 28th dpi, corresponding to the adult and ML stages of *T. spiralis*, respectively, to undergo the assessment criteria [35]. The antitrichinellosis efficacy was assessed via parasitological, ultrastructural, biochemical, and histopathological studies (Fig. 1).

### Prophylactic and therapeutic activity against experimental trichinellosis

#### Parasitological study

##### Adult count

On the 5th dpi, the small intestine of the infected mice was longitudinally opened, washed with phosphate buffer saline, and fragmented into small pieces. After 2 h of incubation at 37°C, the adult worms were collected and microscopically counted [35].

##### Larval count

On the 28th dpi, encysted ML were retrieved by artificial digestion and counted microscopically as previously described [35].

Parasite burden reduction was calculated according to the following equation:

$$\begin{aligned} & \text{Percentage reduction}\left( \%\text{R}\right) \\ &\quad= 100 - \left[ \frac{Mean\:count\:in\:treated\:subgroup}{Mean\:count\:in\:non{\text{-}}treated\:control} \times 100\right] \end{aligned}$$

#### Ultrastructural study

The harvested adult worms and ML from infected non-treated mice (Group III) and infected therapeutic subgroups (Va, Vb, and Vc) were fixed in cold 2.5% buffered glutaraldehyde phosphate, dehydrated, and examined under scanning electron microscopy (SEM) (JEOL JSM, IT200, Japan) [32].

#### Biochemical study

Colorimetric measurement of Malondialdehyde (MDA) and reduced glutathione (GSH) were assayed in sera of mice in all studied groups using commercial kits (CAT. No. MD2529 and GR2511, Bio-Diagnostic, Egypt) according to the manufacturer´s instructions.

#### Histopathological study

One cm segments of jejunal tissue from infected groups (III, IV, and V) were taken on the 5th dpi, fixed in 10% formalin, processed, and embedded in paraffin blocks. Paraffinized sections were stained with hematoxylin and eosin (H&E) and examined microscopically. Ten low-power fields (LPFs) were examined per mouse for the detection of intestinal inflammatory cellular infiltrate, damage, and goblet cell density [31]. Jejunal inflammatory cellular infiltration was determined using a score system from 0 to 4 (0, normal cellular pattern; 1, scattered lamina propria inflammatory cellular infiltrate; 2, numerous lamina propria inflammatory cellular infiltrate; 3, confluent inflammatory cellular infiltrate extending to submucosa; and 4, transmural inflammatory cellular infiltrate). Jejunal tissue damage was evaluated by a scoring system ranging from 0 to 4 (0, normal architectural pattern with normal villous to crypt ratio; 1, minimal villous shortening and broadening with minimal crypt hyperplasia; 2, mild villous shortening and broadening with mild crypt hyperplasia; 3, obvious villous shortening and broadening with obvious crypt hyperplasia; and 4, extensive mucosal damage and extension through deeper structures of bowel wall. Jejunal goblet cell density was determined as mean goblet cell number per 10–15 enterocytes as follows: goblet cell depletion (< 1), average goblet cells (= 1), and goblet cell hyperplasia (> 1) [31, 35]. On the 28th dpi, diaphragms retrieved from infected groups (III, IV, and V) were fixed, processed, embedded in paraffin blocks, sectioned, and stained with H&E. Thedensity of ML and inflammatory cell infiltration were microscopically determined in ten LPFs. ML density was scored as follows: score 1, less than 5 ML per LPF; score 2, 5–10 ML per LPF; and score 3, more than 10 ML per LPF. Inflammatory cell infiltration surrounding the encysted ML was recorded as follows: 1, mild; 2, moderate; and 3, intense infiltrate [35, 36].

### Statistical analysis of the data

Result values were analyzed using the IBM SPSS software package version 20.0. (Armonk, NY: IBM Corp). The normality of continuous data was assessed by the Shapiro-Wilk test. One-way ANOVA test was used for comparing normally distributed quantitative variables of different studied subgroups. Post Hoc test (Tukey) was used for pairwise comparison between each subgroup. The level of significance was judged at 5%.

## Results

### Characterization of CWP and TWH

#### Free amino acids analysis

**Fig. 2:**
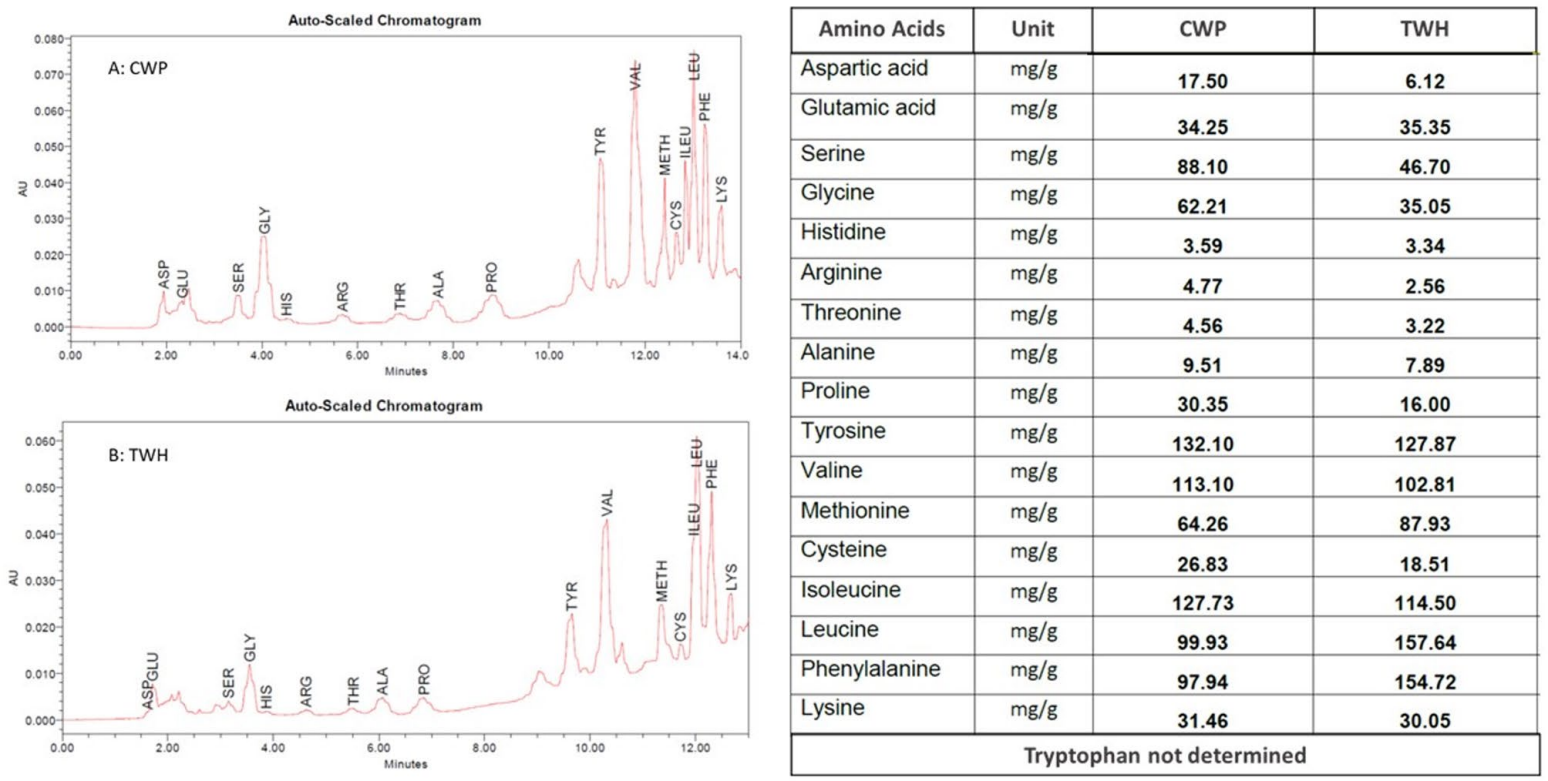
Amino acid profile of CWP and TWH: (**A**) Chromatogram of CWP; (**B**) Chromatogram of TWH; (**C**) Amino acid content of CWP and TWH in mg/g

As illustrated in Fig. 2, the ratios of essential to total amino acids in CWP and TWH were 0.57 and 0.69, respectively. Tryptophan was not determined because it was destroyed during the acid hydrolysis reaction. CWP was highly enriched in essential amino acids, encompassing hydrophobic (isoleucine and valine) and positively charged (lysine) amino acids. Compared to CWP, TWH contained an outstandingly higher concentration of hydrophobic (leucine and phenylalanine) and sulfur-containing (methionine). Additionally, amino acid profiling of CWP and TWH compromised important non-essential amino acids such as hydrophobic (tyrosine and proline), sulfur-containing (cysteine), and negatively charged (glutamic and aspartic) amino acids.

#### Antioxidant activity assay

**Fig. 3:**
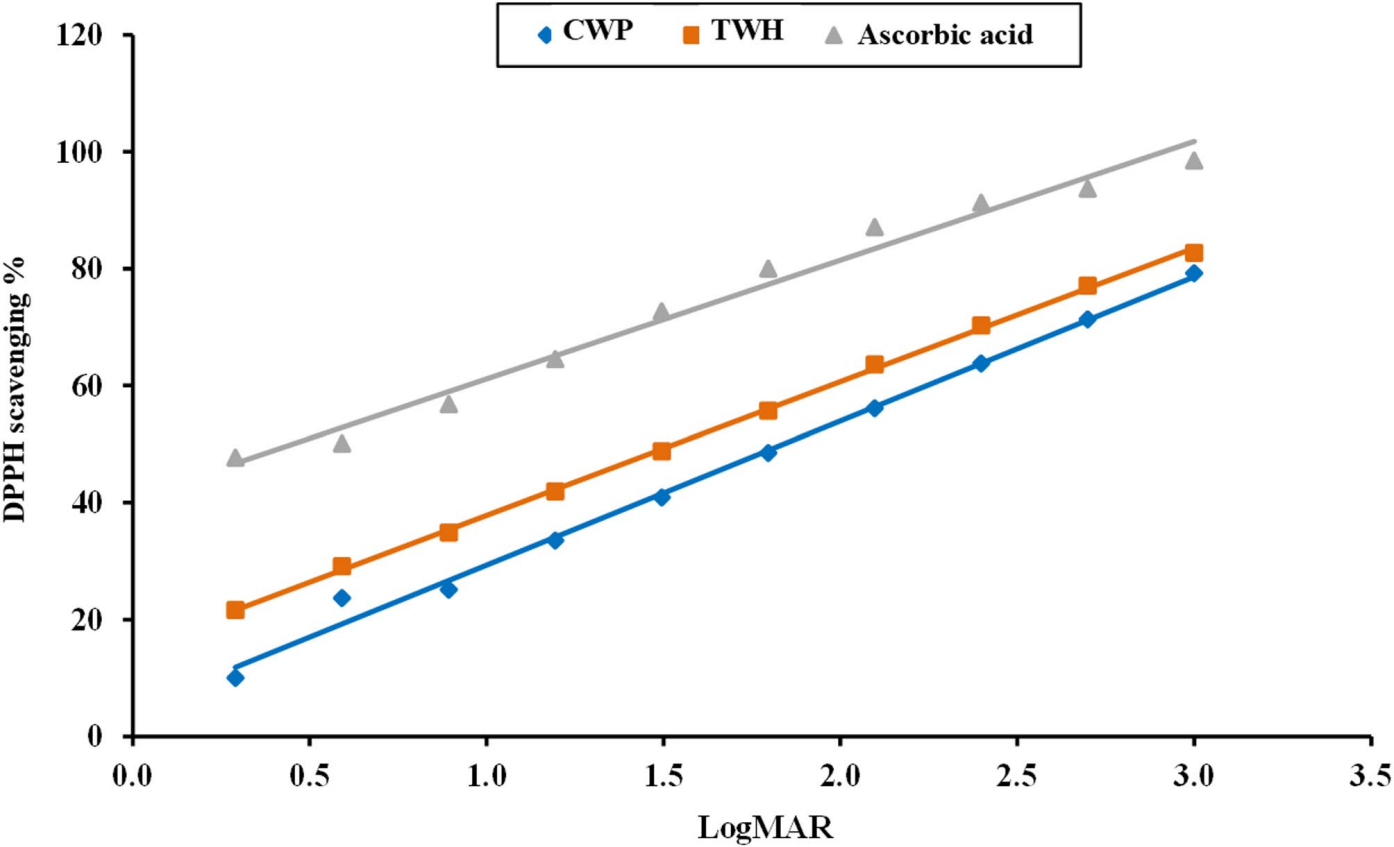
Log dose inhibition curve of the percentage of DPPH free radical scavenging activity of CWP and TWH

As shown in Fig. 3, the scavenging effect on DPPH radicals was higher in TWH (82.7%) when compared to CWP (79.2%). The IC_50_ of TWH was 34.16% and this value was mostly doubled to 69.12% in CWP.

### Prophylactic and therapeutic activity against experimental trichinellosis

#### Parasitological study

##### Adult count

**Fig. 4:**
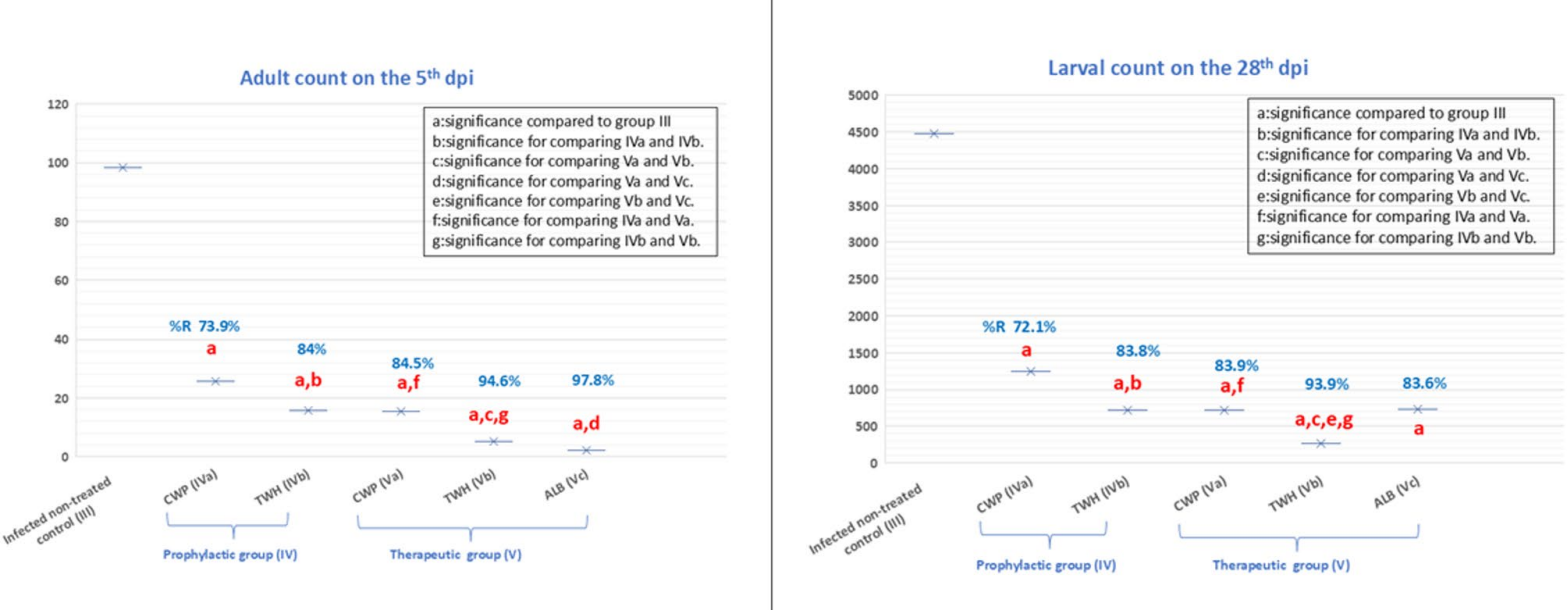
The effect of CWP, TWH, and ALB on parasitic count among the different infected groups

As depicted by Fig. 4, a statistically significant reduction in the mean count of adult worms retrieved on the 5th dpi from the intestine of infected mice either prophylactically or therapeutically-treated was observed compared to infected non-treated control (Group III) ( *p *< 0.001). In prophylactically-treated mice with CWP (Subgroup IVa) and TWH (Subgroup IVb), a statistically significant reduction in the mean adult worm load of 73.9% and 84%, respectively, was reported in favor of TWH (*p* < 0.05). In therapeutic group (V), comparing the effect of CWP and TWH together, a statistically significant difference in the mean adult count of 84.5%R and 94.6%R, respectively, was observed in favor of TWH (*p* < 0.05). While the comparison between CWP and TWH with ALB showed that only ALB was superior to CWP, with a statistically significant difference (*p* < 0.05). When comparing the prophylactic and therapeutic efficacy of either CWP (IVa and Va) or TWH (IVb and Vb), a statistically significant difference in mean adult worm count was noted in favor of the therapeutic ones (*p* < 0.05).

##### Larval count

As shown in Fig. 4, a statistically significant reduction in the mean ML load collected on 28th dpi of infected mice either prophylactically or therapeutically-treated was recorded compared to infected non-treated control (Group III) (*p * < 0.001). In prophylactically-treated mice with CWP (Subgroup IVa) and TWH (Subgroup IVb), a statistically significant reduction in the mean ML count of 72.1% and 83.8%, respectively, was reported in favor of TWH (*p* < 0.05). In therapeutic group (V), when comparing the effect of CWP and TWH together, a statistically significant difference in the mean ML count of 83.9%R and 93.9%R, respectively, was observed in favor of TWH (*p* < 0.05). While the comparison between CWP and TWH with ALB showed that only TWH was superior to ALB, with a statistically significant difference (*p* < 0.05). Interestingly, the comparison between prophylactic and therapeutic efficacy of either CWP (IVa and Va) or TWH (IVb and Vb) revealed a statistically significant superior reduction in mean ML count, favoring the therapeutic ones (*p* < 0.05).

#### Ultrastructural study

**Fig. 5:**
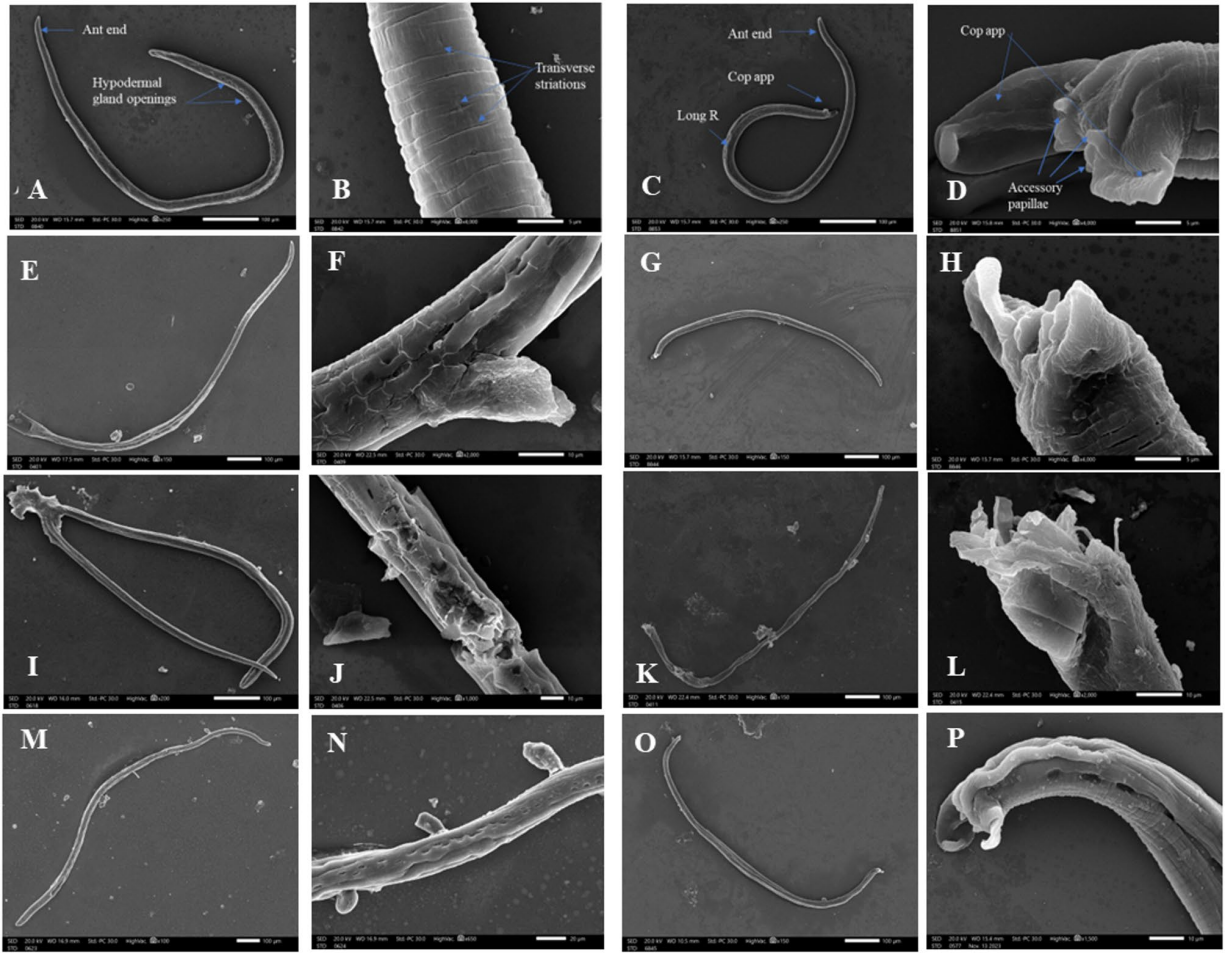
SEM of *T. spiralis* adult worms collected from small intestine of infected non-treated (Group III) and therapeutically-treated mice (Group V): (**A–D**) Adult worms collected from infected non-treated mice (Group III); (**A**) Adult female worm showing a slender anterior cylindrical body and prominent hypodermal gland openings extending to its thicker posterior end (x250); (**B**) the annulated mid body showing transverse cuticular striations with interposed hypodermal gland openings (x4000); (**C**) adult male worm possessing a shorter body with longitudinal ridges ending with a pair of copulatory appendages (x250); (**D**) caudal end of male worm showing copulatory organ formed of two prominent copulatory appendages and four accessory papillae (x4000). (**E–P**) Adult worms recovered from infected therapeutically-treated mice (Group V): (**E–H**) Adult worms collected from CWP-treated mice (Subgroup Va); (**E**) Stretched female worm with cuticular cracking, epidermal sloughing and ruptured posterior end (x150); (**F**) mid body demonstrating epidermal sloughing and cuticular fissuring (x2000); (**G**) male with multiple surface blebs and annulation loss (x150); (**H**) posterior end showing deflated copulatory appendage of male worm (x4000). (**I–L**) Adult worms retrieved from TWH-treated mice (Subgroup Vb); (**I**) Female worm showing deformed body with massive bursting (x200); (**J**) severely degenerated mid body in the form of extended sloughing zones and cavitations (x1000); (**K**) male worm showing severe body disfigurement and erosions (x150); (**L**) ruptured swollen posterior end of male worm with disfigured copulatory appendages (x2000). (**M–P**) Adult worms collected from ALB-treated mice (Subgroup Vc); (**M**) Elongated female worm possessing almost smooth cuticle with multiple blebs and cauliflower masses (x100); (**N**) multiple cuticular cauliflower masses with epidermal sloughing (x650); (**O**) male possessing scattered fungating masses (x150); (**P**) corrugated collapsed posterior end of male worm (x1500)

As illustrated in (Fig. 5), female adult worms collected from infected non-treated control mice (Group III) were cylindrical, tapering anteriorly with a prominent stylet at the cephalic dome (Fig. 5A). Its body possessed transverse cuticular striations perpendicular to longitudinal ridges and evident hypodermal gland openings extending to its posterior end (Fig. 5B). While male worms had shorter annulated bodies ending with a pair of prominent copulatory protuberances and four ancillary papillae (Fig. 5C, D).

On the other hand, female adults treated with CWP (Subgroup Va) were stretched, imparting a thinner body with surface cracking, sloughed detached epidermis, and ruptured posterior end (Fig. 5E, F). Male worms exhibited multiple surface blebings and erosions with loss of cuticular anulations (Fig. 5G). In addition, deflated intact copulatory appendages were evident posteriorly (Fig. 5H). Females treated with TWH (Subgroup Vb) possessed severely deformed, discontinuous bodies along with worm bursting (Fig. 5I). The cuticular degenerative changes were manifested as extended zones of sloughing, disintegration, and cavitations exposing underlying structures (Fig. 5J). Male worms were disfigured with a ruptured cuticle (Fig. 5K), and a featureless, distorted copulatory organ was observed caudally (Fig. 5L). Meanwhile, adult female worms treated with ALB (Subgroup Vc) showed cuticular smoothness associated with multiple surface blebs and cauliflower masses (Fig. 5M, N). Male worms exhibited a smooth, flattened midbody with scattered fungating masses (Fig. 5O) and a corrugated, collapsed posterior end (Fig. 5P).

**Fig. 6:**
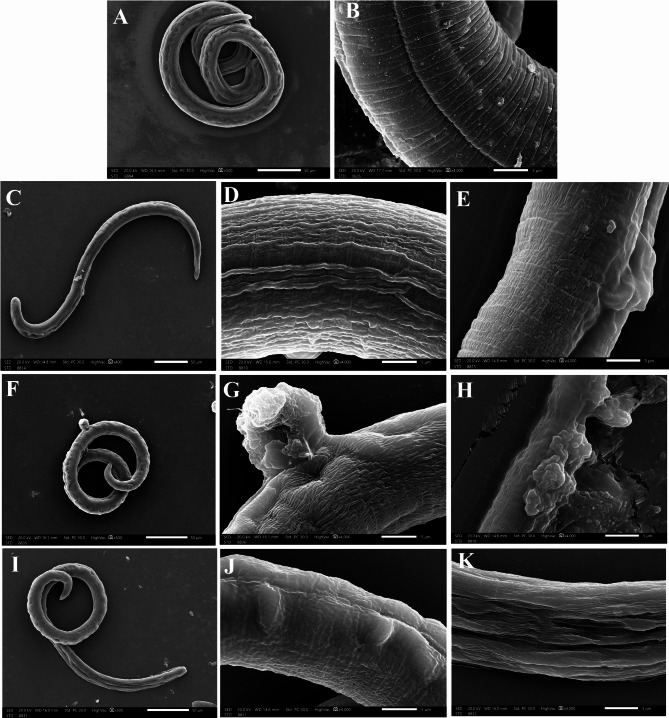
SEM of *T. spiralis* ML collected from infected non-treated (Group III) and therapeutically-treated mice (Group V): (**A–B**) ML retrieved from infected non-treated mice (Group III); (**A**) Normal ML showing idiosyncratic coiling of anteriorly tapered body and evident hypodermal gland openings (x500); (**B**) mid body showing shallow longitudinal groove perpendicular to the annulated cuticle (x4000). (**C–E**) ML collected from CWP-treated mice (Subgroup Va); (**C**) uncoiled ML with widened hypodermal gland openings (x400); (**D**) extensive furrowing of the longitudinal grooves (x4000); (**E**) cuticular blebs and protrusion (x4000). (**F–H**) ML retrieved from TWH-treated mice (Subgroup Vb); (**F**) Loosely coiled ML showing noticeable surface erosions and cauliflower mass (x500); (**G**) ulcerating cauliflower mass along with widened hypodermal gland openings (x4000); (**H**) bursting body extravasating the underlying ML content (x4000). (**I–K**) ML collected from ALB-treated mice (Subgroup Vc); (**I**) Loosely coiled ML lost its annulations with deepening of longitudinal grooves (x500); (**J**) multiple surface blebs on smooth cuticles (x4000); (**K**) Multiple longitudinal cuticular gaps (x4000)

Regarding ML collected from infected non treated control mice (Group III), they possessed cylinderical bodies tapering anteriorly, preserved hypodermal gland openings and idiosyncratic coiling behavoir (Fig. 6A). Longitudinally shallow grooves were allocated perpendicular to the annulated cuticle (Fig. 6B). Recovered ML from CWP-treated subgroup (Va) lost their coiling pattern with widened openings of the hypodermal glands (Fig. 6C). Their cuticle lacked the transverse striations with extensive longitudinal cuticular furrowing (Fig. 6D) associated with surface blebs and protrusions (Fig. 6E). Meanwhile, loosely coiled ML retrieved from TWH-treated subgroup (Vb) showed cuticular erosions, loss of annulations, widened longitudinal furrows and cauliflower masses (Fig. 6F, G). Some areas of the cuticle were ruptured, extravasating the internal contents (Fig. 6H). On the other hand, in ALB-treated group (Subgroup Vc), collected ML showed widening of hypodermal gland openings as well as loss of cuticular annulation (Fig. 6I). Multiple blebs and vesiculations were observed in some parts of the cuticle (Fig. 6J), while others were associated with multiple longitudinal cuticular gapings (Fig. 6K).

#### Biochemical study

##### Malondialdehyde level

Serum MDA is a reliable marker of lipid peroxidation insult induced by both intestinal and muscular stages of *T. spiralis* infection. In the non-infected treated group (II), either CWP, TWH, or ALB, there was a statistically nonsignificant difference in the mean serum level of MDA in comparison to the non-infected non-treated control (Group I) (Table 1).

**Table 1:** The effect of CWP, TWH and ALB on the serum MDA level in nmol/ml among all studied subgroups compared to their controls

Non-infected groups	Non-treated control (I)		CWP-treated (IIa)		TWH-treated (IIb)		ALB-treated (IIc)		F (*p*)
Mean ± SD	1.83 ± 0.19		1.86 ± 0.12		1.89 ± 0.23		1.96 ± 0.49		0.223 (0.879)
Median (Min. – Max.)	1.8 (1.6–2.1)		1.9 (1.6–2)		1.9 (1.5–2.1)		1.9 (1.5–2.9)		

**Infected** **groups**	**Infected** **non-treated** **control** **(III)**	**Prophylactic group** **(IV)**			**Therapeutic group** **(V)**				**F (p)**
		**CWP** **(IVa)**		**TWH** **(IVb)**	**CWP** **(Va)**	**TWH** **(Vb)**		**ALB** **(Vc)**	
** 5th dpi**									1475.391^*^ (< 0.001^*^)
Mean ± SD	17.1 ± 0.6	4.5 ± 0.3		3.9 ± 0.2	3.8 ± 0.3	3.1 ± 0.1		2.3 ± 0.4	
Median (Min. – Max.)	17.1 (16.4–17.8)	4.5 (4.2–5)		3.8 (3.6–4.2)	3.8 (3.3–4.2)	3.1 (2.9–3.2)		2.4 (1.9–2.9)	
p_0_		< 0.001^*^		< 0.001^*^	< 0.001^*^	< 0.001^*^		< 0.001^*^	
Significance	p_1_ = 0.048^*^, p_2_ = 0.012^*^, p_3_ < 0.001^*^, p_4_ = 0.019^*^, p_5_ = 0.015^*^, p_6_ = 0.004^*^								
** 28th dpi**									
Mean ± SD	21.1 ± 0.8	6.8 ± 0.3		6.1 ± 0.2	4.8 ± 0.2	2.5 ± 0.3		3.2 ± 0.4	1682.298^*^ (< 0.001^*^)
Median (Min. – Max.)	21 (20.3–22.4)	6.8 (6.5–7.2)		6.1 (5.8–6.3)	4.9 (4.5–5.1)	2.5 (2.2–3)		3.1 (2.9–3.7)	
p_0_		< 0.001^*^		< 0.001^*^	< 0.001^*^	< 0.001^*^		< 0.001^*^	
Significance	p_1_ = 0.045^*^, p_2_ < 0.001^*^, p_3_ < 0.001^*^, p_4_ = 0.048^*^, p_5_ < 0.001^*^, p_6_ < 0.001^*^								

F: for One way ANOVA test, Post Hoc Test (Tukey) for pairwise comparison

p_0_: p value for comparison between control group and each treated subgroup

p_1_: p value for comparing IVa and IVb; p_2_: p value for comparing Va and Vb

p_3_: p value for comparing Va and Vc; p_4_: p value for comparing Vb and Vc

p_5_: p value for comparing IVa and Va; p_6_: p value for comparing IVb and Vb

*: Statistically significant at *p* ≤ 0.05

In infected groups on the 5th dpi, both prophylactic and therapeutic groups (IV and V) showed a statistically significant decrease in the mean serum MDA level compared to infected non-treated control (Group III) (*p* < 0.001). There was a statistically significant difference between CWP and TWH, either prophylactically (Subgroups IVa and IVb) or therapeutically-treated (Subgroups Va and Vb), in favor of TWH (*p* < 0.05). While the comparison between CWP and TWH with ALB in the therapeutic group (V) revealed a statistically significant difference in favor of ALB (*p* < 0.05). There was a statistically significant difference in the mean serum MDA levels between prophylactic and therapeutic subgroups, either treated with CWP or TWH, in favor of the therapeutic subgroup (*p* < 0.05) (Table 1).

On the 28th dpi, a statistically significant reduction in the mean serum MDA level was recorded in prophylactic and therapeutic groups (IV and V) compared to infected non-treated control (Group III) ( *p* < 0.001). There was a statistically significant difference between CWP and TWH, either prophylactically (Subgroups IVa and IVb) or therapeutically-treated (Subgroups Va and Vb), in favor of TWH (*p* < 0.05). While the comparison between CWP and TWH with ALB, only TWH was superior, with a statistically significant difference (*p* < 0.05). A statistically significant difference in the mean serum MDA levels was recorded between prophylactic and therapeutic subgroups, either treated with CWP or TWH, in favor of the therapeutic subgroup (*p* < 0.001) (Table 1).

##### Reduced glutathione level

Serum GSH level is a reliable indicator of antioxidant activity against oxidant-mediated damage generated by *T. spiralis* infection. In the non-infected treated group (II), either CWP, TWH, or ALB, there was a statistically nonsignificant difference in the mean serum level of GSH in comparison to the non-infected non-treated control group (I) (Table 2).

**Table 2:** The effect of CWP, TWH and ALB on the serum GSH level in mg/dl among all studied subgroups compared to their controls

Non-infected groups	Non-treated control (I)		CWP-treated (IIa)		TWH-treated (IIb)		ALB-treated (IIc)		F (*p*)
Mean ± SD	2.74 ± 0.09		2.76 ± 0.08		2.75 ± 0.09		2.74 ± 0.09		2.397 (0.098)
Median (Min. – Max.)	2.74 (2.6–2.9)		2.7 (2.7–2.9)		2.8 (2.6–2.8)		2.7 (2.6–2.9)		

**Infected** **groups**	**Infected** **non-treated** **control** **(III)**	**Prophylactic group** **(IV)**			**Therapeutic group** **(V)**				**F (p)**
		**CWP** **(IVa)**		**TWH** **(IVb)**	**CWP** **(Va)**	**TWH** **(Vb)**		**ALB** **(Vc)**	
**5th dpi **									403.343^*^ (< 0.001^*^)
Mean ± SD	1.5 ± 0.1	7.6 ± 0.7		8.9 ± 0.3	8.3 ± 0.3	9.5 ± 0.5		2.7 ± 0.3	
Median (Min. – Max.)	1.5 (1.4–1.6)	7.6 (6.8–8.6)		8.9 (8.4–9.2)	8.4 (7.7–8.6)	9.5 (9–10.2)		2.7 (2.3–3.1)	
p_0_		< 0.001^*^		< 0.001^*^	< 0.001^*^	< 0.001^*^		< 0.001^*^	
Significance	p_1_ < 0.001^*^, p_2_ < 0.001^*^, p_3_ < 0.001^*^, p_4_ < 0.001^*^, p_5_ = 0.079, p_6_ = 0.100								
** 28th dpi **									
Mean ± SD	1.2 ± 0.1	2.4 ± 0.5		2.7 ± 0.4	7 ± 0.3	8.8 ± 0.3		2.7 ± 0.3	444.512^*^ (< 0.001^*^)
Median (Min. – Max.)	1.2 (1–1.3)	2.5 (1.8–2.9)		2.8 (2.2–3.2)	7 (6.6–7.4)	8.8 (8.4–9.3)		2.8 (2.3–3.1)	
p_0_		< 0.001^*^		< 0.001^*^	< 0.001^*^	< 0.001^*^		< 0.001^*^	
Significance	p_1_ = 0.693, p_2_ < 0.001^*^, p_3_ < 0.001^*^, p_4_ < 0.001^*^, p_5_ < 0.001^*^, p_6_ < 0.001^*^								

F: for One way ANOVA test, Post Hoc Test (Tukey) for pairwise comparison

p_0_: p value for comparison between control group and each treated subgroup

p_1_: p value for comparing IVa and IVb; p_2_: p value for comparing Va and Vb

p_3_: p value for comparing Va and Vc; p_4_: p value for comparing Vb and Vc

p_5_: p value for comparing IVa and Va; p_6_: p value for comparing IVb and Vb

*: Statistically significant at *p* ≤ 0.05

In infected groups on the 5th dpi, both prophylactic and therapeutic groups (IV and V) showed a statistically significant increase in the mean serum GSH level compared to infected non-treated control (Group III) (*p* < 0.001). A statistically significant difference between CWP and TWH, either prophylactically (Subgroups IVa and IVb) or therapeutically-treated (Subgroups Va and Vb), was recorded in favor of TWH (*p* < 0.001). Compared to ALB in the therapeutic group (V), both CWP and TWH were superior with a statistically significant difference (*p* < 0.001). There was no statistically significant difference in the mean serum GSH levels between prophylactic and therapeutic subgroups treated with either CWP or TWH (*p* > 0.05) (Table 2).

On the 28th dpi, a statistically significant increment in the mean serum GSH level was recorded in prophylactic and therapeutic groups (IV and V) compared to the infected non-treated control (Group III) ( *p*< 0.001). On comparing CWP and TWH, either prophylactically (Subgroups IVa and IVb) or therapeutically-treated (Subgroups Va and Vb), only a statistically significant difference was recorded in the therapeutic group favoring TWH (*p* < 0.001). Compared to ALB in the therapeutic group (V), both CWP and TWH were superior with a statistically significant difference (*p* < 0.001). There was a statistically significant difference in the mean serum GSH levels between prophylactic and therapeutic subgroups, either treated with CWP or TWH, in favor of the therapeutic subgroup (*p* < 0.001) (Table 2).

#### Histopathological study

**Fig. 7:**
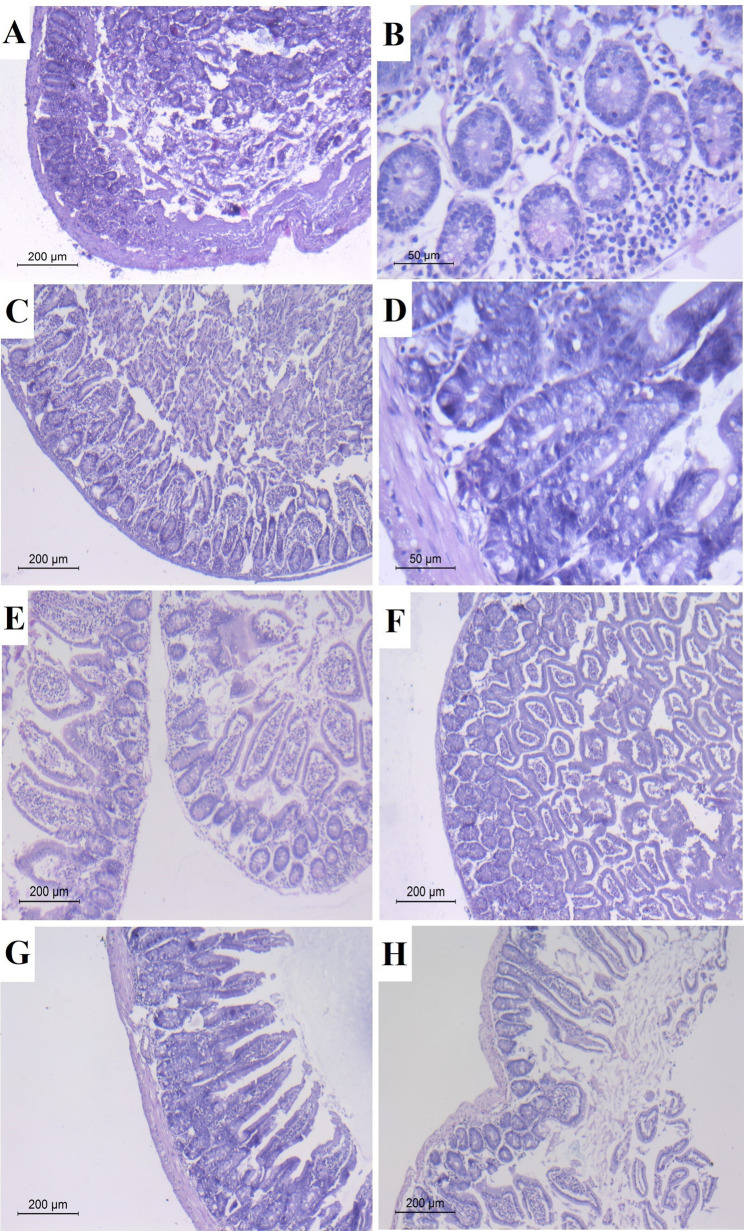
Histopathological findings of H&E-stained jejunal sections of different infected groups: (**A–B**) sections of infected non-treated mice (Group III); (**A**) showing numerous inflammatory cellular infiltrates in the lamina propria (score 2), sloughed mucosa and obvious crypt hyperplasia (score 3) (x100); (**B**) goblet cell hyperplasia (x400). (**C–D**) sections of CWP-pretreated mice (Subgroup IVa); (**C**) showing moderate inflammatory infiltrates in lamina propria (score 2), minimal shortening of villi and crypt hyperplasia (score 1) (x100); (**D**) an average number of goblet cells (x400). (**E**) Sections of TWH-pretreated mice (Subgroup IVb) showing scattered inflammatory cellular infiltrate in lamina propria (score 1) with normal villous to crypt ratio (score 0) and an average goblet cell number (x100). (**F**) Sections of CWP-treated mice (Subgroup Va) recorded scanty cellular infiltration of lamina propria (score 1), minimal shortening of villi and crypt hyperplasia (score 1) with an average goblet cell count (x100). (**G**) Sections of TWH-treated mice (Subgroup Vb) revealing normal cellular pattern (score 0), villous to crypt ratio (score 0), and an average goblet cell number (x100). (**H**) Sections of ALB-treated mice (Subgroup Vc) revealing scattered cellular infiltration in lamina propria (score 1) in the absence of mucosal damage (score 0) and an average goblet cell count (x100)

As depicted in Figs. 7 and 9, jejunal sections of the infected non treated control mice (Group III), showed numerous inflammatory cells occupying the lamina propria (score 2) with sloughed overlying epithelial lining, obvious crypt hyperplasia (score 3) (Fig. 7A) and goblet cell hyperplasia (Fig. 7B). In the prophylactic group, intestinal sections of CWP-pretreated mice (Subgroup IVa) revealed a moderate inflammatory infiltrate mainly in the lamina propria (score 2) along with minimal villous shortening, broadening and hyperplastic crypts (score 1) (Fig. 7C) attaining an average number of goblet cells (Fig. 7D). Whereas the TWH-pretreated mice (Subgroup IVb) demonstrated a significant improvement in the intestinal pathology in the form of scattered lamina propria inflammatory cellular infiltrate (score 1) without any mucosal damage manifested as a normal villous to crypt ratio (score 0) along with an average goblet cell number (Fig. 7E). In the therapeutic group, intestinal sections of mice treated with CWP (Subgroup Va) recorded scanty scattered inflammatory cellular infiltration of lamina propria (score 1) and minimal villous shortening and crypt hyperplasia (score 1) associated with an average goblet cell count (Fig. 7F). While the intestinal sections of mice treated with TWH (Subgroup Vb) restored normal mucosal architecture revealing neither inflammatory cells (score 0) nor signs of mucosal damage (score 0) with an average goblet cell number (Fig. 7G). Whereas the intestinal sections of mice treated with ALB (Subgroup Vc) revealed a scattered inflammatory cellular infiltrate in lamina propria (score 1) in the absence of mucosal damage (score 0) and with an average goblet cell number (Fig. 7H).

**Fig. 8:**
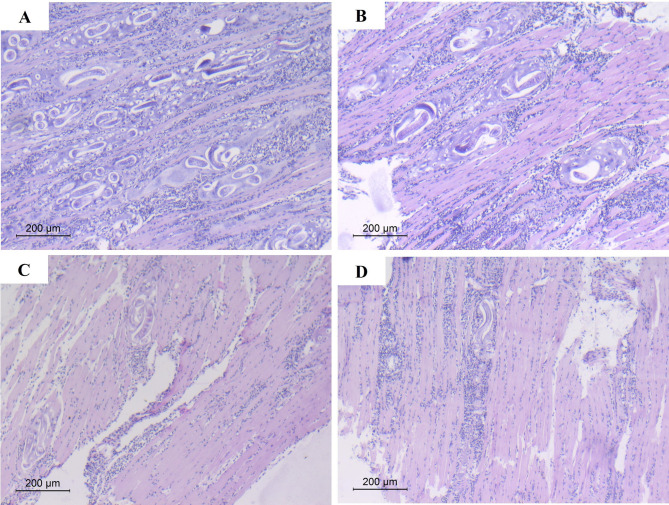
Histopathological findings of H&E-stained diaphragmatic muscle sections of different infected groups: (**A**) sections of infected non-treated mice (Group III) showing highly densely populated ML (score 3) surrounded by intense chronic inflammatory infiltrate (score 3) (x100). (**B**) Sections of CWP-pretreated mice (Subgroup IVa) revealing moderate ML density (score 2) surrounded by moderate inflammatory infiltration (score 2) (x100). (**C**) Sections of CWP-treated mice (Subgroup Va) showing low ML density (score 1) with mild inflammatory cellular infiltrate (score 1) (x100). (**D**) Sections of ALB-treated mice (Subgroup Vc) showing low ML density (score 1) with moderate cellular infiltrate (score 2) (x100)

**Fig. 9:**
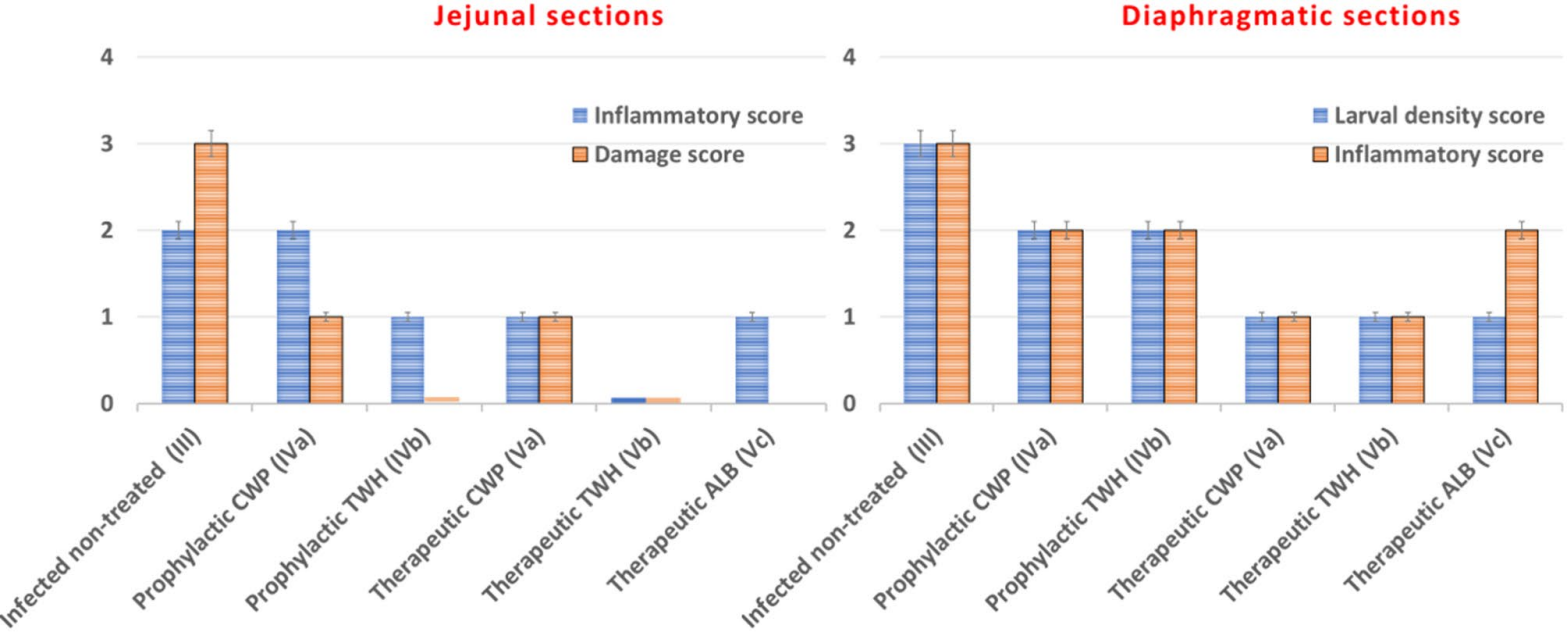
Scoring of histopathological findings in H&E-stained jejunal and diaphragmatic sections among the different infected groups

As illustrated in Figs. 8 and 9, diaphragmatic muscle sections of the infected non-treated control mice (Group III) showed densely populated ML (score 3) surrounded by an intense chronic inflammatory infiltrate (score 3) (Fig. 8A). In the prophylactic group, muscular sections of both CWP and TWH-pretreated mice (Subgroups IVa and IVb) revealed a moderate ML density (score 2) surrounded by a moderate inflammatory infiltrate (score 2) (Fig. 8B). In the therapeutic group, diaphragmatic sections of both CWP and TWH-treated mice (Subgroup Va and Vb) showed a low density of ML (score 1) with a mild inflammatory cellular infiltrate (score 1) (Fig. 8C). Whereas sections of ALB-treated mice (Subgroup Vc) recorded a low ML density (score 1) with a moderate cellular infiltrate (score 2) (Fig. 8D).

## Discussion

Trichinellosis, a globally re-emerging meat-borne parasitosis, may occasionally cause disability-adjusted life years or even death [37]. The gold standard drug, ALB, has a profound potent effect on the intestinal adult stages of *T. spiralis* [3]. Nevertheless, overcoming the ongoing unsolved pharmacological flaws of ALB is considered the forthcoming challenge. Taking these considerations into account, the utilization of a natural, safe, affordable, and efficient antioxidant nutraceutical as a potential prophylactic and therapeutic candidate for trichinellosis is an undeniable, credible approach.

The promising anti-*Trichinella* activity of dromedary CWP and its trypsinized hydrolysate in the present study was based merely on their amino acids and bioactive peptides attributes [16]. It is reasonable to consider that several factors account for such a considerable reduction in worm burden. Firstly, the currently used CWP is rich in proline amino acid, which is capable of killing the pathogens by interacting with the ribosomes and subsequently disrupting their protein synthesis [38]. Secondly, the presence of the privileged antipathogenic iron-binding glycoprotein, lactoferrin, which competes for iron, an essential element for survival of *T. spiralis* in the body of its mammalian host [39]. Building on previously published data, the obvious reduction in worm burden could be explained by considering the accelerated expulsion of adult worms upon treatment with either CWP or TWH. Our deduction could be explained by those who proved that the monosaccharide content of glycomacropeptides, which constituted about 15–20% of the total CWP, acts as an antiadhesive agent against enteric pathogens [40]. It is plausible that the potential anti-*Trichinella* prophylactic activity of either CWP or TWH could be attributed to the good bioavailability of their bioactive peptides after oral administration, as their elimination half-lives ranged from minutes up to a few hours [41].

The superior antiparasitic performance of TWH than CWP was ascribed to its higher antioxidant potential. The magnificent potential of TWH was owed to the release of encrypted inactive antioxidant low molecular weight bioactive peptides containing antioxidant amino acids trapped within the CWP, with more accessibility to neutralize free radicals and make them innocuous. This is in harmony with published data documenting significant improvement in free radical scavenging antioxidant activity of whey proteins after enzymatic hydrolysis by exposing the buried amino acids within parent proteins and increasing availability of hydrogen ions [42]. Our findings agreed with those who reported that subsequent proteolysis of CWP with trypsin increased the antimicrobial activity of hydrolysate by threefold due to its higher pronounced antioxidant activity than CWP (Salami et al., 2010). It was reasonable to speculate that the higher antiparasitic potential of TWH is dependent on its specialized amino acid profile [22]. The higher content of methionine in TWH is capable of accelerating the inhibition of DNA synthesis of the parasite [43]. Additionally, the hydrophobic amino acids (leucine, phenylalanine, and methionine) are salient and predominant in TWH. Our results were consistent with those who provided a preponderance of evidence that the predominance of hydrophobic amino acids was responsible for the highest antifungal activity of CWP and TWH [43].

In the same vein, ultrastructural alterations in worms recovered from trichinous mice treated with either CWP or TWH endorsed the parasitological results. The more severe ultrastructural deformities observed in worms treated with TWH attest to the prodigiously distinguished parasitological results. This can be ascribed to the trypsin’s greater affinity to hydrolyse the peptide bond of CWP at hydrophobic residues [43]. The predominant hydrophobic amino acids, methionine, leucine, and phenylalanine, in TWH electrostatically bound to the cuticle of *T. spiralis*, ultimately, led to its cleavage, pore formation, and profound destruction [25]. It is noteworthy that the significant reduction in adult worm count conformed with the evident ultrastructural disruption of adult worms caused by the inhibitory binding of ALB to the cuticular β-tubulin [44]. Notwithstanding, the less pronounced efficacy of ALB was observed in ALB-treated ML, as they were protected by a capsule, which prevented them from direct exposure to the anthelmintic ALB [4].

Uncontrolled oxidant-mediated damage produced by the host’s defence response and *Trichinella* is responsible for the detrimental pathogenesis of trichinellosis [45]. Serum MDA concentration is a reliable marker of radical-induced cytotoxic oxidative damage in the host along the course of trichinellosis infection [46]. The increase in serum MDA level in the *T. spiralis*-infected mice implies that the released endogenous antioxidants were not sufficient to overcome the uncontrolled cytotoxic oxidation reaction. Our results were also in agreement with those who reported a sharp increase in the serum level of MDA in *T. spiralis*-infected rats up to 4 weeks [47]. Therefore, it is reasonable to speculate that the mitigation of oxidative stress by natural antioxidants, CWP or TWH, would help the host to defend against the oxidative damage induced by *T. spiralis*.

The increment in serum antioxidant GSH level of infected mice treated with either CWP or TWH, witnessed in the current study, aimed to limit overwhelming oxidative stress. Consistent with their scavenging effect on DPPH radicals, the implausible antioxidant potential of CWP was more enhanced upon tryptic hydrolysis. Both provide a high content of sulphur comprising amino acids, methionine and cysteine, that effectively promote a strong radical scavenger antioxidant activity by acting as substrates for GSH synthesis [48]. Moreover, the presence of specific amino acids with ring structures, such as histidine, tyrosine, and proline, provides insight into the potent antioxidant activity, as these rings act as powerful proton donors to free radicals [40]. TWH is rich in the negatively charged acidic amino acid, glutamic acid, which possesses surplus electrons that play roles in oxidative stress cessation [49]. Likewise, a previous study substantiated that CWP was a strong natural antioxidant as it increased glutathione levels [50]. This is corroborated by those who recorded that CWP after trypsin treatment exhibited a higher pronounced antioxidant activity than CWP [51].

The histopathological changes detected in the host along the course of trichinellosis are the devastating toll of imbalanced oxidant/antioxidant status [52]. In the current study, restoration of intestinal histological mucosal structure in infected mice after oral administration of CWP and TWH could be explained in the light of the evident reduction in adult worm load, as well as their spectacular antioxidant potential. Our results conformed to those who reported intact mucosal structure and function upon lactoferrin treatment during *T. spiralis*-induced enteritis in mice [39]. The degree of trichinellosis severity is proportionate to the number of ML [2]. The skeletal muscular damage was induced not only by the presence of *T. spiralis* ML itself but also by phagocytic production of high levels of free radicals, aiming for the elimination of the parasite [53]. In the present study, the higher antioxidant potential of TWH improved the histopathological effect of muscle tissue induced by the infection. Besides, the predominance of leucine amino acid in TWH holds the secret behind the restoration of muscular integrity as it plays a vital role in the regulation of muscle protein production, metabolism, and reversible phosphorylation of mRNA [54].

## Conclusion

Given the above-presented data, the superiority of TWH over CWP as an oral natural antioxidant nutraceutical in inducing a spectacular multistage activity and its ability to ameliorate trichinellosis-associated pathology can be concluded. This study is the first evidence on the potential prophylactic and therapeutic efficacy of dromedary CWP and its trypsinized hydrolysate against experimental intestinal and muscular trichinellosis. It drives home the point that the high antioxidant potential of TWH gives ground to such promising antitrichinellosis activity. Further substantiations are needed to investigate their efficacy during the chronic phase of infection and explore the underlying pharmacodynamic and kinetic profiles, and immunological basis of CWP and its tryptic hydrolysate.

## Data Availability

Data is provided within the manuscript.

## Abbreviations

% R: Percentage reduction
A: Absorbance
ALB: Albendazole
CWP: Camel whey protein
dpi: Days post infection
DPPH: 1,1-diphenyl-2-picrylhydrazyl
GSH: Reduced glutathione
H&E: Hematoxylin and eosin
HPLC: High performance liquid chromatography
IC_50_%: Inhibitory concentration of 50%
LPFs: Low power fields
MDA: Malondialdehyde
ML: Muscle larva
SEM: Scanning electron microscopy
*T. spiralis*: *Trichinella spiralis*
TWH: Tryptic whey hydrolysate
UV: Ultraviolet
